# Predicting the Electronic Absorption Band Shape of Azobenzene Photoswitches

**DOI:** 10.3390/ijms24010025

**Published:** 2022-12-20

**Authors:** Ricard Gelabert, Miquel Moreno, José M. Lluch

**Affiliations:** 1 Departament de Química, Universitat Autònoma de Barcelona, Bellaterra, 08193 Barcelona, Spain; 2 Institut de Biotecnologia i de Biomedicina, Universitat Autònoma de Barcelona, Bellaterra, 08193 Barcelona, Spain

**Keywords:** photoswitch, azobenzene derivatives, mammal optical window, absorption spectrum, absorption band shape, molecular dynamics simulations

## Abstract

Simulations based on molecular dynamics coupled to excitation energy calculations were used to generate simulated absorption spectra for a family of halide derivatives of azobenzene, a family of photoswitch molecules with a weak absorption band around 400–600 nm and potential uses in living tissue. This is a case where using the conventional approach in theoretical spectroscopy (estimation of absorption maxima based on the vertical transition from the potential energy minimum on the ground electronic state) does not provide valid results that explain how the observed band shape extends towards the low energy region of the spectrum. The method affords a reasonable description of the main features of the low-energy UV-Vis spectra of these compounds. A bathochromic trend was detected linked to the size of the halide atom. Analysis of the excitation reveals a correlation between the energy of the molecular orbital where excitation starts and the energy of the highest occupied atomic orbital of the free halide atom. This was put to the test with a new brominated compound with good results. The energy level of the highest occupied orbital on the free halide was identified as a key factor that strongly affects the energy gap in the photoswitch. This opens the way for the design of bathochromically shifted variants of the photoswitch with possible applications.

## 1. Introduction

Photoswitches have become a valuable asset in many applied fields, ranging from material science and nanotechnology to biochemistry and biomedicine and in general, wherever it is necessary to control the activation of a molecular device with spatial precision and selectivity in the least invasive way. The simplest concept of a photoswitch consists of a small molecule that can exist in two different forms or “states” that can interconvert by means of absorption of electromagnetic radiation of adequate energy. This interconversion process is usually a light-induced isomerization, most often an E/Z isomerization of a double bond, even though other processes should in principle also be usable (e.g., a tautomerization). A good photoswitch needs to undergo the photoisomerization process quickly; both forms need to be photostable, and most notably, the two directions of the photoisomerization (E → Z and Z → E) should require electromagnetic radiation of sufficiently different energies such that they should be individually addressable. Often, the “switching off” step can also proceed thermally if the process is fast enough.

E/Z isomerizations carry with them an important change in shape and size of the photoswitch. Photopharmacology is a new paradigm in pharmacology that makes use of this property to cause a photoswitchable drug to change between two forms, only one of which is pharmacologically active [1,2]. This permits delivery of drugs that can later be activated using light with temporal and spatial precision. This process is especially applicable to drugs that have many undesired side effects, for instance in chemotherapy [3,4]. In addition, clever design of the substitutions on the photoswitch makes it possible to attach it, for instance, to protein receptor channels and once there, to use light to trigger the switch and activate the channel with spatial and temporal resolution at will. In this sense, it has been used to control the biological activity of living organisms [5,6,7].

These applications require that the photoswitch be activated whilst in the living organism. Mammal living tissue is partly transparent to electromagnetic radiation only between approximately 650 nm and 1100 nm. This means that for optimal use, a photoswitch should activate (photoisomerize) when the light of the wavelength within this tiny window is used. A standard E/Z isomerization can be triggered by absorption of a photon if it promotes an electron from an occupied π molecular orbital (MO) to an unoccupied $\pi^{*}$ MO localized on the double bond that must isomerize. This turns the double bond into a single bond with essentially free rotation. However, usual $\pi \pi^{*}$ transitions occur at wavelengths around 300 nm or less except in extended conjugated systems and are thus far from adequate, aside from the risks involved for living tissue when irradiation happens at these wavelengths.

**Figure 1:**
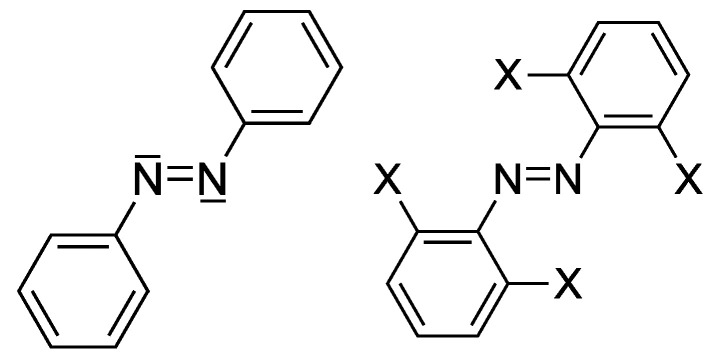
Left: azobenzene (AB); Right: basic structure of tetra-*ortho*-substituted azobenzenes. In both cases, the *E* isomer is depicted.

An alternative to alkene isomerization is the use of derivatives of azobenzene (AB, Figure 1) [8]. ABs have a double bond and two lone electron pairs, a fact that opens the possibility of promoting an electron from a non-bonding orbital (*n*) to the empty $\pi^{*}$ MO that severs the double bond. As *n* electrons are usually higher in energy than π electrons the energy of the excitation is reduced. ABs show the intense $\pi \pi^{*}$ transition around 320 nm, but a weaker band corresponding to the $n\pi^{*}$ transition is found substantially redshifted at around 440 nm. Besides their spectroscopic qualities, ABs have excellent properties that have turned them into the photoswitch motif of choice: easy synthesis, their photoisomerization is reversible, and both forms are relatively photostable. However, with regards to their use in living tissue, their excitation energy is not optimal as the excitation band is still well outside the optical window.

Different strategies have been explored to overcome this difficulty. Biphotonic (2P) transitions have been used to induce the same transition via a double-photon absorption event, which effectively requires each photon to have half the energy or twice as much wavelength, which brings it neatly into the transparency window, in this way avoiding the use of harmful radiation. This has been achieved and put in practice in living tissue with good results [7]. However, technical requirements are quite demanding, and 2P absorption of azobenzenes is quite weak, which explains the enduring interest in finding a way to continue whenever possible using one-photon (1P) techniques.

Considerable effort has been put into trying to push the $n\pi^{*}$ band into this optical window. Substitution of the basic AB motif with electron-rich substituents with lone pairs has managed to shift the absorption wavelength bathochromically. Hecht and co-workers studied the family of tetra-*ortho*-fluoroazobenzenes [9,10], which display $n\pi^{*}$ maxima of absorption at 458 nm. Woolley and co-workers developed tetra-*ortho*-methoxyazobenzenes [11,12] and tetra-*ortho*-chloroazobenzenes [13,14], where the wavelength shifts further to about 480 nm. Excitation of the $n\pi^{*}$ band achieved photoisomerization despite the weakness of the band. Feringa et al. devised optimized synthetic procedures for this kind of molecules, which had otherwise quite a convoluted synthesis [15]. These modified ABs manage to shift the excitation band bathochromically with respect to the original compound but do not move the absorption band fully inside the bio-optical window.

Nevertheless, one possibility to continue using these conventional 1P excitations relies on the fact that absorption bands extend over a wide range of wavelengths on both sides of the peak, in some cases attaining widths of about 200 nm. Thus, irradiation many tens of nms towards the lower energy side of the peak might still trigger the same photoisomeridation with one photon at a fraction of the efficiency. Woolley and co-workers determined effective photoisomerization of tetra-*ortho*-substituted ABs with bulky electron-rich substituents (X=OMe, Cl, Br, see Figure 1) with wavelengths between 630 nm and 650 nm, despite the fact that the absorption band peak is found around 480 nm [13]. This was used to control the structure of linked peptides using red light [13]. Feringa and co-workers managed to control the activation of antibacterial activity of trimethoprim (an antibacterial drug) with red light, linking it to tetra-*ortho*-substituted azobenzenes with X=F, Cl (see Figure 1) [3]. Irradiation at 652 nm induced very slow E→Z photoisomerization and faster velocities when irradiation took place at 527 nm. In general, photoswitching at wavelengths far from the peak absorption is a valid approach.

Computational and theoretical chemistry provide many tools that can be valuable assets to interpret the outcome of experiments as a first step to design systems with improved qualities. The issue we want to explore here is: What are the possibilities that computational chemistry offers to predict the general (complete) line shape of the absorption spectrum?

Intensity of one-photon absorption (or emission) processes depends on the square of the so-called transition dipole moment $\langle \Psi_{i}|\mu |\Psi_{f}\rangle$, where $\Psi_{i}$ and $\Psi_{f}$ are the wave functions of the initial and final states involved in the transition, and μ is the dipole moment operator. Under the Born–Oppenheimer approximation, these wave functions can be written as a product of an electronic and a nuclear wave function, such that

(1) $$\langle \Psi_{i}\left|\mu \right|\Psi_{f}\rangle =\langle \psi_{v_{i}}|\mu_{if}|\psi_{v_{f}}\rangle .$$

In Equation (1), the bracket operates on nuclear coordinates only, $\psi_{v_{i}}$ and $\psi_{v_{f}}$ denote the rovibrational wave functions of the initial and final states, and $\mu_{if}$ is the electronic transition dipole moment

(2) $$\mu_{if}=\langle \psi_{\mathrm{el},i}\left|\mu \right|\psi_{\mathrm{el},f}\rangle$$

where the bracket operates over electronic coordinates this time. Conventional absorption spectra involve transitions from a ground vibronic state to all rovibrational states in the excited electronic state, which could make this calculation complicated due to the large number of states involved. Usually the Franck–Condon principle can be applied, which greatly simplifies this calculation. The excitation is considered to be so fast that the nuclear configuration does not change while it takes place [16,17]. Mathematically, this makes $\mu_{if}$ a constant, and the intensity of the transition depends on the overlap of the vibrational states (Franck–Condon factors). This is the basis of the vertical transition rule, which in short equates the maximum of the absorption band to the excitation energy computed at the geometry of the minimum in the initial electronic state. This is, in general, a precise approximation except for weakly allowed or dipole-forbidden transitions.

The vertical transition rule does not provide information about the shape of the absorption band, nor about its width. If irradiation at energies different from that of the peak is of interest, it becomes necessary to obtain the full absorption band, which might imply varying degrees of sophistication. It might suffice to compute the rest of Franck–Condon factors (overlaps of vibrational functions) if the Franck–Condon principle is applicable. If not, Equation (2) will have to be used where the electronic transition dipole moment is no longer a constant and depends on the nuclear coordinates. This can be performed expanding $\mu_{if}$ as a Taylor series in terms of the normal mode distance to a reference point. In this context, the Franck–Condon approximation is just the truncation at the constant term (zero-order series). The Herzberg–Teller approximation is based on a linear expansion (first-order series) [18]. This procedure can continue expanding the Taylor series as needed. However, this is a laborious process.

Li and Truhlar recently devised a model that could obtain the overall extent of the absorption band exclusive of the detailed vibrational structure [19] derived from the work of Heller and co-workers on time-dependent absorption spectroscopy [20,21,22,23]. The method assumes that the transition dipole moment does not vary with geometry in the area relevant to spectroscopy and that both potential energy surfaces (ground and excited state) are quadratic and in fact, only differ on the position of the minima and have identical Hessians. The expressions derived for the band shape are based on the assumption that the minima of both ground and excited states are close in configurational space (close enough to be within the range of a Taylor expansion of the potential truncated at second order).

It is implicit in both approaches that they will not work well for any absorption process where the excited state minimum differs substantially from the ground state structure. Computation of all Franck–Condon factors or inclusion of configurational dependence on the electric dipole transition moment will suffer on top if highly excited vibrational states are required. This is the case because anharmonicity is guaranteed to be present in highly excited vibrational states and anharmonic calculations are usually not affordable for many-atom systems. The alternative in these cases is what has been known as the ensemble method, where all vibronic excitations are computed from a set of structures spanning the dominion of the nuclear wave functions generated by different means, most commonly by a classical trajectory [24,25,26]. This approach has the advantage that it can be carried out in the presence of solvent molecules using computationally efficient methods.

In this paper, we present an application of the ensemble method using molecular dynamics simulations to compute the shape of the electronic absorption spectrum of a series of azobenzene derivatives in DMSO. The results will be used to analyze the causes that affect the shape and extent of the absorption bands and propose yet another variant with an absorption band that is shifted to lower energies.

## 2. Results and Discussion

In what follows, we will describe various simulations used to elucidate the nature of the accessible excited states of several azobenzene derivatives. We will also compute simulations of their absorption spectra based on molecular dynamics simulations to sample the configurational space open to these photoswitches in solution at a given temperature. A detailed account of the protocols used can be found in the Materials and Methods section.

We begin this study determining the conventional vertical excitation energies for the series of *E* tetra-*ortho*-substituted azobenzenes (X=H, F, Cl: see Figure 1) as follows. We carry out a geometry optimization of the ground electronic state of each molecule both in the gas phase and in the presence of solvent (DMSO) using the CPCM method. We then compute vertical excitation energies in both cases. We focus on the characteristics of the two lowest singlet excited states, $S_{1}$ and $S_{2}$. Results are shown in Table 1.

The only *E* isomer of these azobenzene derivatives that is planar is azobenzene itself (X=H), where the optimized structure belongs to the $C_{2h}$ symmetry point group. In the fluorinated and chlorinated derivatives, and arising likely from steric interactions originating in these bulkier substituents, planarity cannot be achieved, and the structures have both phenyl rings out of plane to some extent, reducing the symmetry of the minimum to only $C_{2}$. The differences in symmetry of the minima will have some effects on the optical accessibility of certain excited states, as will be discussed below.

The lowest-lying excited states appear in two spectral regions, 2.6–2.8 eV and 3.9–4.5 eV, depending on the substituent X and the presence or not of the solvent. $S_{1}$ appears to be relatively insensitive to both factors, but $S_{2}$ shows a noticeable hypsochromic effect when halogens are present in the structure. For $S_{2}$, the effect of the solvent is apparently larger and tends to reduce the excitation energy, indicating that $S_{2}$ has an increased dipole moment with respect to the ground state.

**Figure 2:**
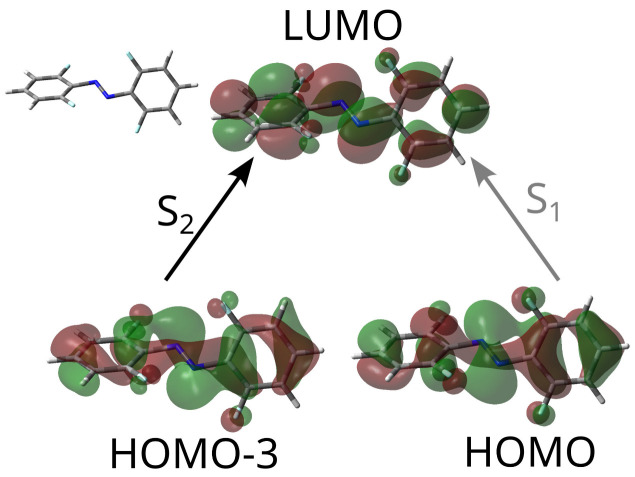
Molecular orbitals involved in the excitations giving rise to excited singlet states $S_{1}$ and $S_{2}$ in the case of the substituted azobenzene with X=F. The different intensities of the arrows indicate the magnitude of the transition probabilities from the ground state by means of electromagnetic radiation.

Analysis of the nature of the excited states can be conducted within the configuration interaction framework, analyzing the molecular orbitals involved in the excitation. Figure 2 shows the orbitals involved in the excitations giving rise to $S_{1}$ and $S_{2}$ in the case of the fluorinated derivative. The situation is equivalent in the case of the chlorinated derivative; in both cases, the molecule is not completely planar.

Both $S_{1}$ and $S_{2}$ have in common that the target (end point) of the electronic excitation is actually the LUMO. This orbital is spread all over the molecule, but in the specific region of the azo group, it is locally antisymmetric with respect to the plane of the double bond and has a nodal surface that splits it. Therefore, it can be described as locally $\pi^{*}$ over the region of the double bond.

In both states, the orbital where excitation starts has local π character over each of the phenyl moieties, but in the region on top of the azo group, the character of the MO is actually a mix. It has traits of π character (because if is partly antisymmetric with respect to the double-bond plane, in the sense that the wave function has different signs on both sides of this plane), but the shape of each lobe is different. This “unclear” character with respect to the local double bond plane is a consequence of the fact that the molecule belongs to the $C_{2}$ symmetry point group, and no real symmetry plane containing the full molecule exists. Both excitations, then, break the double bond on the azo group, but they cannot be described either as $\pi \pi^{*}$ or $n\pi^{*}$. What the calculations reveal is that $S_{1}$ is accessed with less probability than $S_{2}$ using electromagnetic radiation as a result of the precise value of the transition dipole moment.

The discussion above is also valid for the substituted azobenzenes with X=F, Cl. An interesting observation can be made about $S_{1}$ for the original *E*-azobenzene. In this case, the molecule is planar and contains a center of inversion. The orbital where excitation starts is of $A_{u}$ symmetry, while the destination orbital is of $B_{g}$ symmetry, and this results in the excitation being optically forbidden (f=0). This shows the risks involved in using the simplified approach based on computing pure vertical transitions from a single preferred structure (ground state minimum). While this approach predicts that the excited state is not accessible by means of a monophotonic excitation, the experimental spectrum of *E*-azobenzene shows two bands, one strong and clearly identified with a $\pi \pi^{*}$ transition around 325 nm (3.8 eV), but also a band about 100 times weaker around 440 nm (2.8 eV) (correspondingly, $n\to \pi^{*}$).

Actually, the molecule is never in the configuration where excitation is formally forbidden, except on average. Molecular vibrations and interaction with the solvent, if present, combine to make the geometry of the molecule visit a certain volume of configurational space that contains (among others) this particular geometry. In this classical picture of the molecule evolving in configurational space, interaction of the molecule with the electromagnetic field can happen in many geometries slightly different from that of the minimum. Excitation energies will depend on the precise geometry, as will the transition probability. In this sense, a “forbidden transition” because the molecule has a $C_{2h}$ geometry will actually be (weakly) allowed because the molecule is caught by the photon while outside of that symmetry.

In order to predict the position and spread of these bands, we implemented an ensemble study of the solvated azobenzenes described. The method relies on a dynamical simulation of each solvated substituted azobenzene molecule in DMSO at constant temperature and pressure. Each of these simulations (one per substituent) is used to sample a series of uncorrelated snapshots of the system, and each of these is used to compute the excitation energies, using electronic embedding to introduce the polarization of the electronic density of the solute by each specific configuration of the solvent molecules.

**Figure 3:**
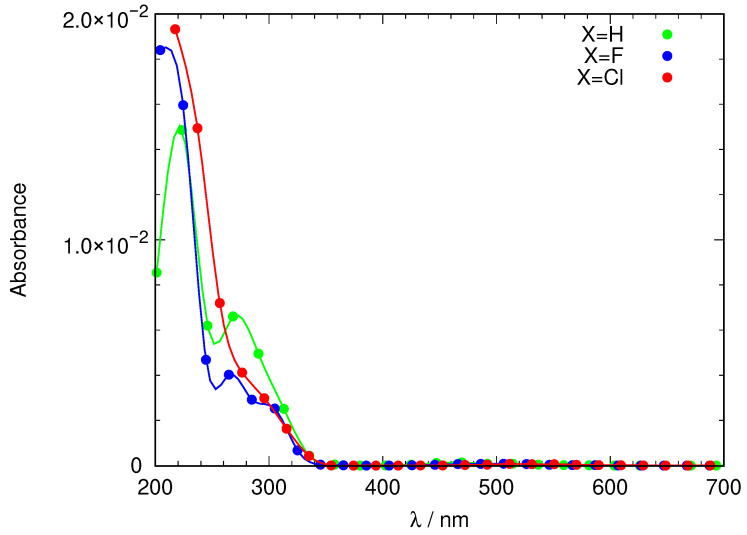
Molecular dynamics simulation of the absorption spectrum of azobenzene derivatives (X=H, F, Cl) using a GAFF2 force-field description of the solute and the solvent. Absorbance is given in arbitrary units.

Azobenzene, its tetra-*ortho*-fluorinated and -chlorinated derivatives and the DMSO solvent molecule were optimized using quantum mechanical electronic structure methods, using the CAM-B3LYP functional and the 6-311+G(d,p) basis set, and the results were fed to the Antechamber program to produce a set of GAFF2 force-field parameters. Using the protocol described in the Materials and Methods section, we prepared three systems consisting of an azobenzene derivative molecule solvated by DMSO using a single capped octahedron cell to generate the PBC. The system was then equilibrated at constant temperature (300 K) and pressure (1 bar), after which it was observed that energy and density were oscillating around a central value. A total of 10 ns of production molecular dynamics were computed. Along the MM/GAFF2 MD simulation, a structure was sampled each 10 ps and used to compute the excitation energies of the 10 lowest-lying excited singlet electronic states. Using the method described in the Materials and Methods section, a simulation of the electromagnetic spectrum was constructed, which is depicted in Figure 3.

**Figure 4:**
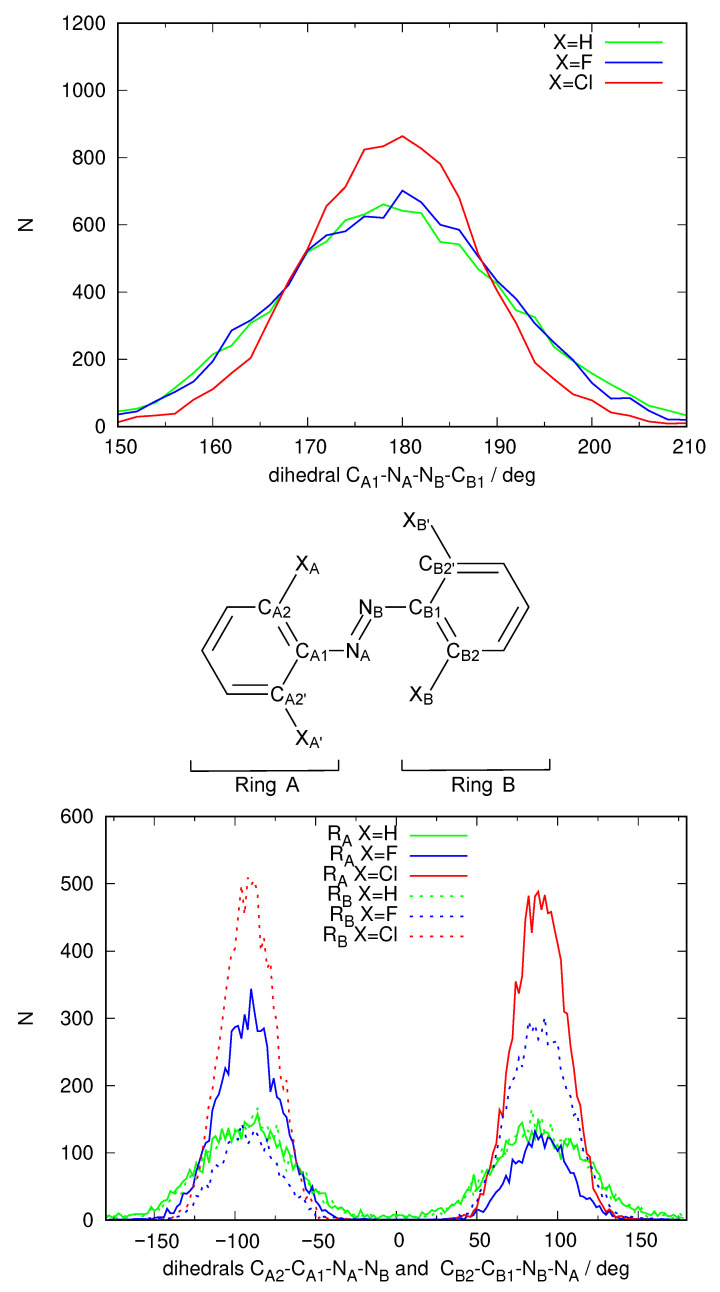
Distribution of the values of specific geometric parameters over the full MD simulations using the GAFF2 force-field parameters used to construct the spectra for the three different azobenzene derivatives with X=H (azobenzene), F and Cl as shown in Figure 3. Labels of the atoms are defined on the structure in the center. Top: distribution of the dihedral defining the *E*-*Z* configuration of the N=N double bond; Bottom: distribution of the dihedral defining the inclination of the two phenyl rings with respect to the azo group.

The spectrum shown in Figure 3 is not completely satisfactory: The band describing access to $S_{1}$ is located roughly between 400 nm and 500 nm and quite weak, as expected, but the main feature, the excitation to $S_{2}$ which ought to be present around 300 nm is unstructured, almost missing, hidden under the towering band peaking around 200–240 nm that corresponds to transitions to other excited states of energy higher than $S_{2}$. An analysis of the structures that form the dynamical simulation reveals an anomaly in the geometries that each solute adopts during the simulation. Figure 4 shows a frequency plot detailing the values of the dihedral angle defining the configuration of the N=N double bond and the orientation of both phenyl rings with respect to the N=N bond. It can be seen that while the *E* configuration of the N=N double-bond remains stable over time, the orientation of the phenyl rings with respect to the N=N double bond is strange. Ror X=H, two peaks at ±90 are observed for each phenyl ring, meaning that both rings can rotate more or less freely around the respective C-N bond but on average adopt a perpendicular conformation with respect to the azo unit. When X=F rotation is more hindered, each ring is found mostly either at +90 or −90. Finally, X=Cl rotation is so hindered that it does not happen during the 10 ns of the simulation. In any case, no structure is captured where these phenyl units are coplanar to the azo group (angles of 0 and ±180). In such an arrangement, the conjugation of the π system cannot take place. The dihedral angle defining the configuration of the N=N double bond is 180 in the gas phase minimum energy structures found using DFT methodology, with only slight deviations for X=F, Cl (177). However, the dihedral angle describing the inclination of the phenyl rings with respect to the N=N double bond are quite different in this case (X=H 0, X=F 36, X=Cl 52). Hence, the simulation puts the phenyl rings completely perpendicular to the azo unit, making even a partial conjugation of the π system impossible.

In summation, this MD simulation does not reproduce the correct geometry of the AB derivatives in solution. The C-N bonds in the AB derivative are treated as pure σ bonds (i.e., bonds with almost free rotation). All of this results in the trajectory visiting the wrong regions of configurational space, where the phenyl rings are perpendicular to the C-N=N-C group.

There are different approaches that can be adopted in order to correct this. A better description of the potential energy landscape of the solute molecule is needed, in particular the degrees of freedom describing the torsion of the phenyl groups with respect to the N=N bond. One could re-parameterize the force field using high-level electronic structure calculations as a reference. While correct, such an approach is length, y and one runs the risk of altering the potential energy of other degrees of freedom unless a large set of structures is computed. A different possibility open for modern molecular dynamics software packages is to carry out the MD simulation using an approximate QM method for a part of the system (here the solute molecule) and to compute its energy on-the-fly along the dynamics. In this case no parameterization is needed, the solute molecule is treated fully quantum-mechanically (albeit at an approximate level), and the effect of the solvent can be introduced using electronic embedding. AMBER can work with QM/MM methods. In particular, the DFT tight binding (DFTB) method is implemented.

**Figure 5:**
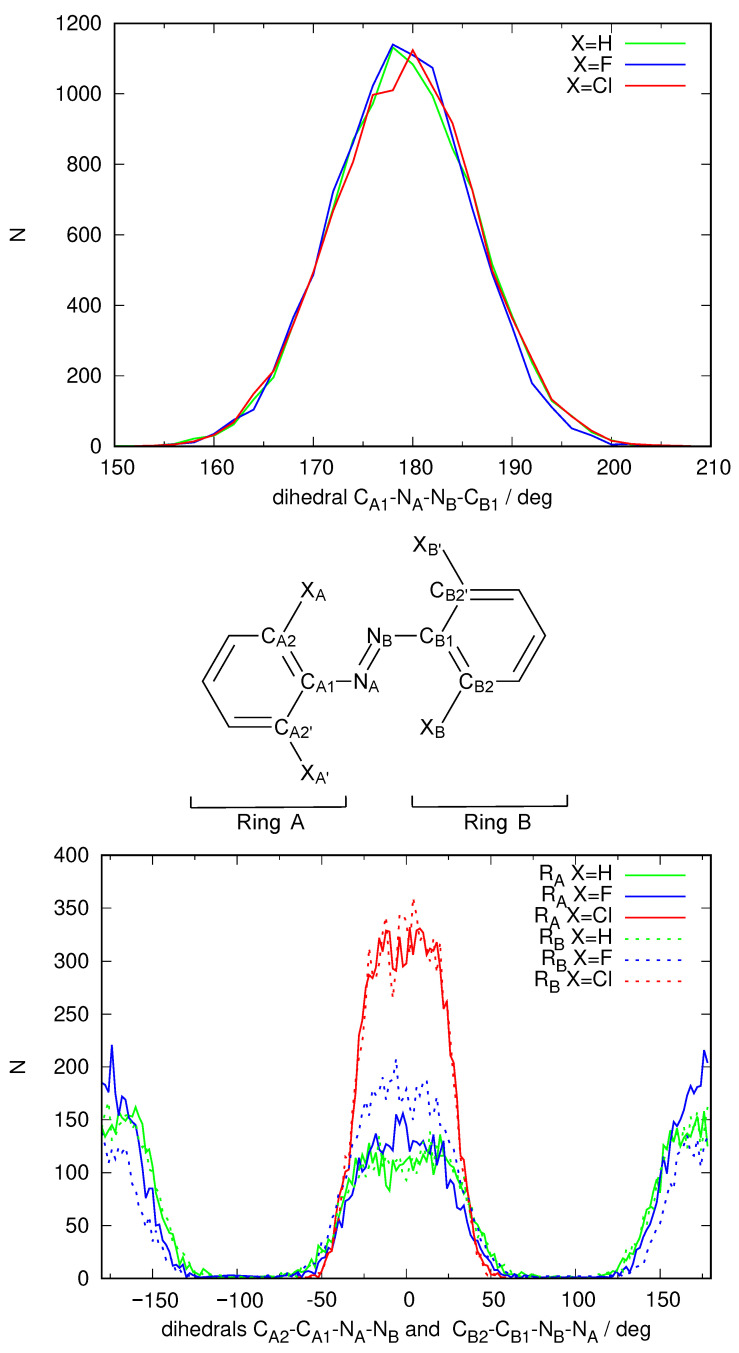
Distribution of the values of specific geometric parameters over the QM/MM MD simulations using a DFTB description of the solute molecules used to construct the spectra for the three different azobenzene derivatives with X=H (azobenzene), F and Cl as shown in Figure 6. Labels of the atoms are defined on the structure in the center. Top: distribution of the dihedral defining the *E*-*Z* configuration of the N=N double bond; Bottom: distribution of the dihedral defining the inclination of the two phenyl rings with respect to the azo group.

We then repeated the 10 ns NPT simulation using a QM/MM scheme, where the solute molecule is described using DFTB and GAFF2 to describe the DMSO solvent molecules. Electronic embedding was used to reproduce the polarization of the solute due to the presence of the solvent molecules. Figure 5 shows the frequency graph for the dihedral angles describing the *E*/*Z* configuration of the N=N double bond and the torsion angle describing the rotation of each phenyl group with respect to the N=N double bond.

The analysis of the configuration of the N=N double bond in the DFTB simulation shows that under this new description of the energetics of the solute, the *E* configuration of the N=N double bond is preserved. In fact, the distribution is narrower around a dihedral value of 180, showing that the N=N double bond is more rigid than in the previous pure MM simulation. More importantly, both phenyl groups are now seen to prefer a coplanar configuration with respect to the N=N bond. The distribution of the corresponding dihedrals (bottom panel of Figure 5) shows a unimodal distribution for X=Cl centered at 0 (which means that the phenyl rings do not manage to rotate at all in the 10 ns that have been simulated). A bimodal distribution can be seen centered at 0 and ±180 for X=H and F with equal areas (which means that each phenyl ring manages to “flip” frequently but stays almost all of the time in a coplanar configuration with the N=N double bond). Hence, the DFTB/MM simulation manages to introduce a strong electronic effect that will enable the electronic conjugation of the π system of the full solute molecule in contrast to the pure MM simulation. This fact is expected to have an effect on the predicted spectrum.

**Figure 6:**
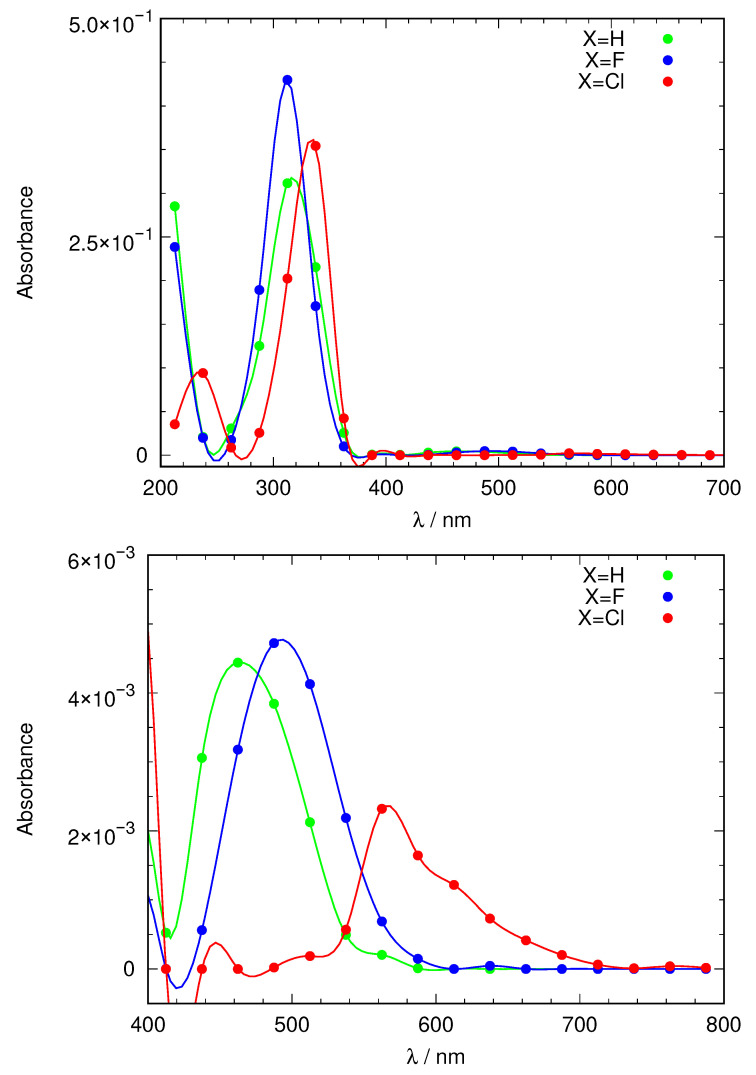
Simulation of the absorption spectra of azobenzene derivatives (X=H, F and Cl) based on a QM/MM molecular dynamics simulation, where the solute is described using DFTB. Top: full range including UV and visible spectra featuring the transition to $S_{2}$; Bottom: visible part of the spectrum highlighting the weak transition to $S_{1}$. Absorbance is given in arbitrary units.

Figure 6 shows the simulation of the UV-Vis spectrum for the azobenzene derivatives with X=H, F and Cl. A startling difference appears in the shape of a band peaking between 260–360 nm depending on the nature of X. This band, which was almost missing in the simulation derived from the MM MD simulation (Figure 3), is intense, and based on the MOs involved in the excitation, it corresponds to excitation to $S_{2}$ in Figure 2.

Figure 6 also shows an enlargement of the weak band, indicating transition to $S_{1}$, which is actually the most interesting one as it is distinctly closer to the “optical transparency window” that would enable efficient use of the photoswitch in living tissue. These bands are substantially weaker than their $S_{2}$ counterparts. Interestingly, their maxima show a distinct bathochromic effect as X goes from H (460 nm) to F (490 nm) and finally to Cl (560 nm). Aside from the noticeable overall effect implying a shift of 100 nm, this is especially remarkable because the values derived from excitation energy calculations, both in the gas phase and DMSO (within CPCM theory), shown in Table 1 reduced this effect to only 11.5 nm and 11.6 nm, respectively, and without a clear trend.

**Figure 7:**
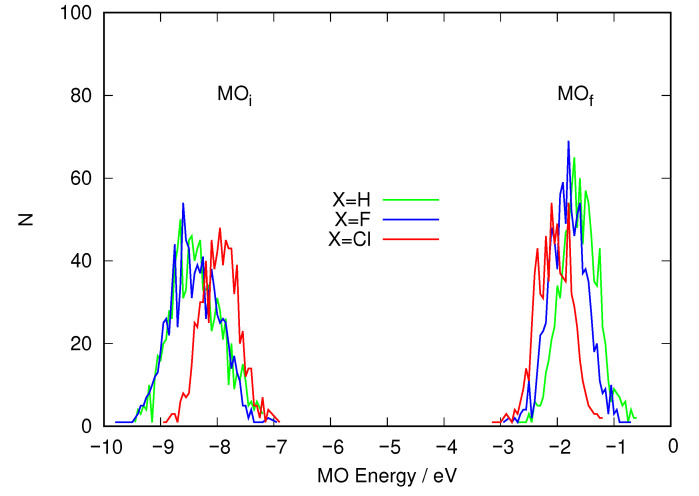
Distribution of the energy of the MOs involved in the excitation that gives rise to $S_{1}$ taken from the snapshots from the QM/MM molecular dynamics simulation where the solute is described using DFTB. MO${}_{i}$ (left) denotes the orbital where the excitation starts and MO${}_{f}$ (right) the orbital where the excitation ends.

To discover what is causing this behavior, we analyzed the excitation energy calculations for the three solute molecules and histogrammed the energies of the two MOs involved. This implies 1000 snapshots for each molecule. The histograms are presented in Figure 7. This graph shows that the MO where excitation starts and the one where it ends show an effect. Along the series X=H→F→Cl, the receiving MO sinks in energy (becomes stabilized) although slightly. The average values go from −1.59 eV (H), −1.78 eV (F) and −1.86 eV (Cl). The largest effect is seen in the MO where excitation starts, however. The values rise this time: −8.34 eV (H), −8.38 eV (F) and −7.78 eV (Cl). It is clear that the strongest effect is in the departing orbital when a heavy halogen atom is considered.

Conventionally, $S_{1}$ is described as a $n\pi^{*}$ excited state and $S_{2}$ as a $\pi \pi^{*}$ excited state. This agrees with the relative intensities of the transitions to both (weak and strong, respectively), as well as the expected ordering of the MOs; *n* (non-bonding) orbitals are described as derived from lone pairs of the atoms and accordingly higher in energy than bonding π MOs. This fact suggests that $n\pi^{*}$ excited states should lie lower in energy than $\pi \pi^{*}$ ones. Even without considering the fluxionality introduced by the dynamics, these molecules do not have a symmetry plane (except for X=H), so the labels used are just approximate in the sense that they refer to the characteristics of the MOs in a local environment to the azo group, as can be seen in Figure 2. In this sense, the departing MO (the *n* part) is not an orbital composed solely from lone pairs. It has substantial contributions on the phenyl groups that are locally π to these rings as well as sizeable contributions on the nitrogen and X atoms. Consequently, its energy must be affected when the nature of X changes. CAM-UB3LYP calculations of the atoms F and Cl reveal that the sHOMO for the former is −15.2 eV and −11.5 eV for the latter. Hence, if the lone pairs of X have an effect on the energy of the HOMO of the azobenzene derivative because they take part in the LCAO describing it, the observed trend would agree with the highest energy electrons of chlorine being higher than those of fluorine. In a rough sense, this agrees with the fact that both atoms belong to different periods. Chlorine involves the 3*p* shell while fluorine only the 2*p* one, lower in energy.

An approximate procedure to assess the statement above was undertaken, as follows: We computed the approximate “weight” of the atomic orbitals involved into the MOs that take part in the excitation to $S_{1}$. The “weight” of AOs from atom α in the *i*th MO is defined as follows:

(3) $$\omega_{\alpha }^{i}=\sqrt{\frac{1}{N_{\alpha }}\sum_{j=1}^{N_{\mathrm{AO}}}{\left|c_{j}^{i}\right|}^{2}\delta_{j\alpha }}$$

where α = N or X, $N_{\alpha }$ is the number of atoms of type α in the solute (four for α = X, two for α = N), the summation runs over all AOs in the molecule, $c_{j}^{i}$ is the LCAO coefficient of the *j*th AO in the *i*th MO, and $\delta_{j\alpha }$ is one if the *j*th AO belongs to an atom of α type and zero otherwise. As a weight, $\omega_{\alpha }^{i}$ is only approximate as the AO set is not orthonormal. As a consequence $\omega_{\alpha }^{i}$ is not necessarily contained in the [0, 1] interval. Its lowest possible value is still 0, and the higher its value, the larger the contribution of AOs on atoms of type α to the MO under scrutiny, *j*. We computed this weight for the excitation to $S_{1}$ using the MOs as computed for the gas phase structures of the corresponding azobenzenes. Results are shown in Table 2.

Table 2 reveals that the nature of the *destination* MO (the LUMO) remains constant when changing the halogen, basically a large participation of the nitrogen atoms (∼0.50) and a small contribution of the AOs of the halogen (∼0.12). This is consistent with the LUMO being described as a $\pi^{*}$ MO with strong character on the N=N moiety. Most of its character is determined by the nitrogen atoms. In what concerns the *departing* MO (the HOMO), it can be seen that the contribution of the AOs based on nitrogen atoms is a little smaller (∼0.42) than in the destination MO, but the contribution of the halogen increases when increasing its atomic number, almost doubling when changing from F to Cl. Aside from orbital symmetry considerations, which should not play a relevant role here as the atoms (and orbitals) involved are similar, the main difference is the energy of the AOs before they mix to form the MO. In the case of X=Cl, the larger involvement of its AOs when compared to F suggests that the resulting MO might have a larger contribution of the chlorine atom’s AOs, and this may increase the energy of the HOMO.

This line of reasoning suggests a mechanism to push the $n\pi^{*}$ ($S_{1}$) band further to longer wavelengths. The next element down the halide series, bromine, fills the 4*p* shell. Even though energy spacing decreases as the main quantum number increases, we find the sHOMO of bromine to be −10.5 eV, so about 1 eV less stable than chlorine’s. This suggests that the azobenzene derivative with X=Br should display the $S_{1}$ excited electronic state shifted bathochromically with respect to the compound with X=Cl because its HOMO, in which the bromine orbitals take part, should be shifted towards higher energies, hence decreasing the HOMO-LUMO energy gap. Indeed, Table 2 shows the approximate weight of the orbitals in the case X=Br. In the single structure analyzed, bromine fits the same tendency described before. The LUMO is approximately unaltered, but the HOMO shows increased mixing of the bromine AOs.

To verify this hypothesis, we started over again with a fourth substituent: X=Br. Moreover, to ensure convergence, this time we extended the production run under constant temperature and pressure to cover 30 ns, and we extended the three previous cases to reach this figure. After the simulation was run, a total of 3000 snapshots were sampled from each and used to compute excitation energies for the lowest-lying excited states.

**Figure 8:**
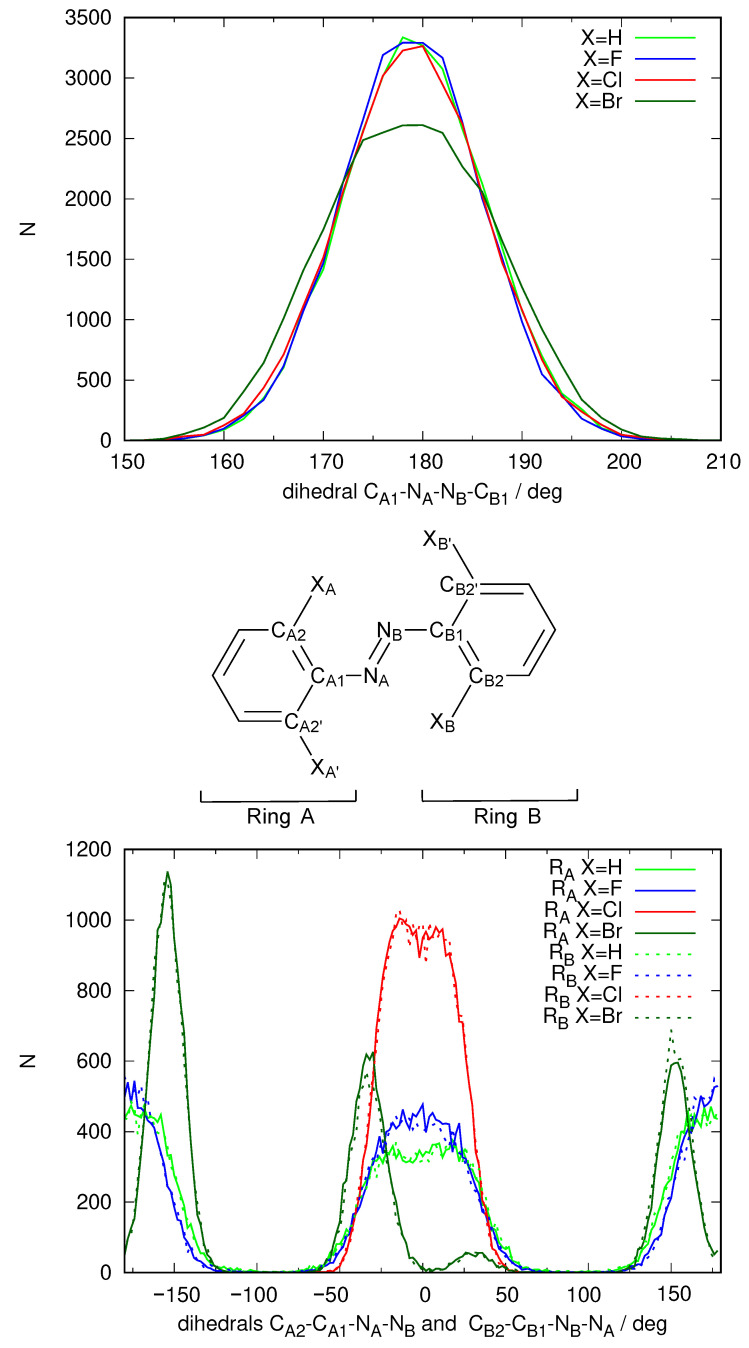
Distribution of specific geometric parameters over the 30 ns QM/MM MD simulation for the four azobenzene derivatives (X=H, F, Cl, Br), where the solute is described using DFTB. Labels of the atoms are defined in the central scheme. Top: distribution of the dihedral defining the *E*-*Z* configuration of the N=N double bond; Bottom: distribution of the dihedral defining the inclination of the phenyl groups with respect to the azo group.

We began the study by analyzing the planarity of the solute molecule in the molecular dynamics simulation. Figure 8 shows the usual selection of dihedrals that help visualizing the coplanarity of the molecule and its phenyl moieties. The distribution of the C${}_{A}$-N${}_{A}$=N${}_{B}$-C${}_{B}$ dihedral indicates a well-preserved *E* configuration of the N=N double bond. However oscillations were observed, and the distribution was slightly less sharp than for the other substituents. The orientation of the phenyl rings presented large differences with respect to the other species studied previously. For X=Br the phenyl rings were never found to be coplanar with the N=N double bond plane. Instead, staggered configurations at ±150 and to lesser extent ±30 were found. Moreover, the areas of the respective maxima of the distributions were unequal. The picture that emerged from these data was one where the phenyl rings could not attain coplanarity with the N=N bond plane, and where a complete flip was difficult (as the areas were of different value). The larger steric hindrance posed by the bulky bromine substituents was very likely the cause for these hindered rotations.

**Figure 9:**
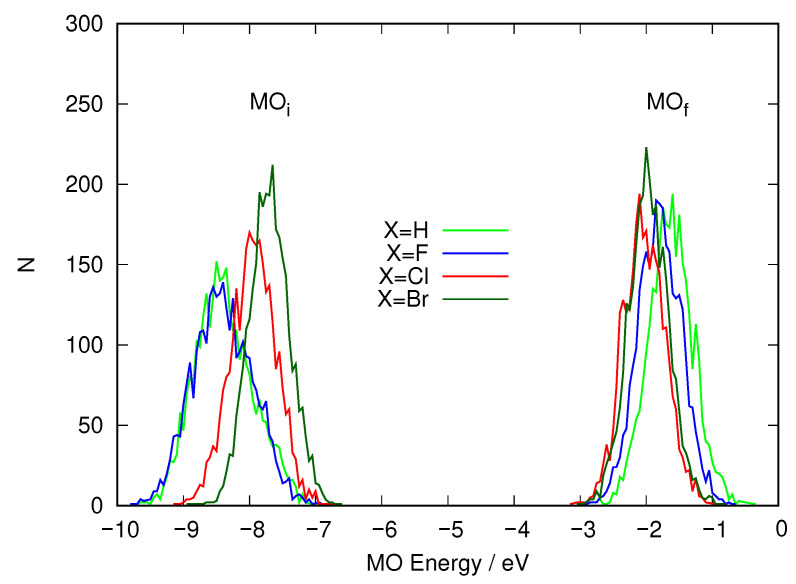
Distribution of the energy of the MOs involved in the excitation that gives rise to $S_{1}$ taken from the snapshots of the 30 ns QM/MM molecular dynamics simulation, where the solute is described using DFTB. MO${}_{i}$ (left) denotes the orbital where the excitation starts and MO${}_{f}$ (right) the orbital where the excitation ends.

What happened to the energies of the MOs involved in the description of the $S_{1}$ excited electronic state? Figure 9 shows the distribution of energy values for the initial and final orbitals for the excitation. This histogram confirms the trends already observed for the two previous halogens. The LUMO energies are distributed almost as in the case of X=Cl. In contrast, when X=Br, the HOMO energies are distributed noticeably to higher energies than in the X=Cl case, which reduces the average excitation energy. Table 3 shows the average values for the energies of these orbitals. It is quite clear that there is a steady trend that reduces the average excitation energy in going along the halogen series F→Cl→Br and that the main cause is an increase of the energy of the MO where excitation starts (the HOMO), connected to the rise in the energy of the atomic orbitals of X.

**Figure 10:**
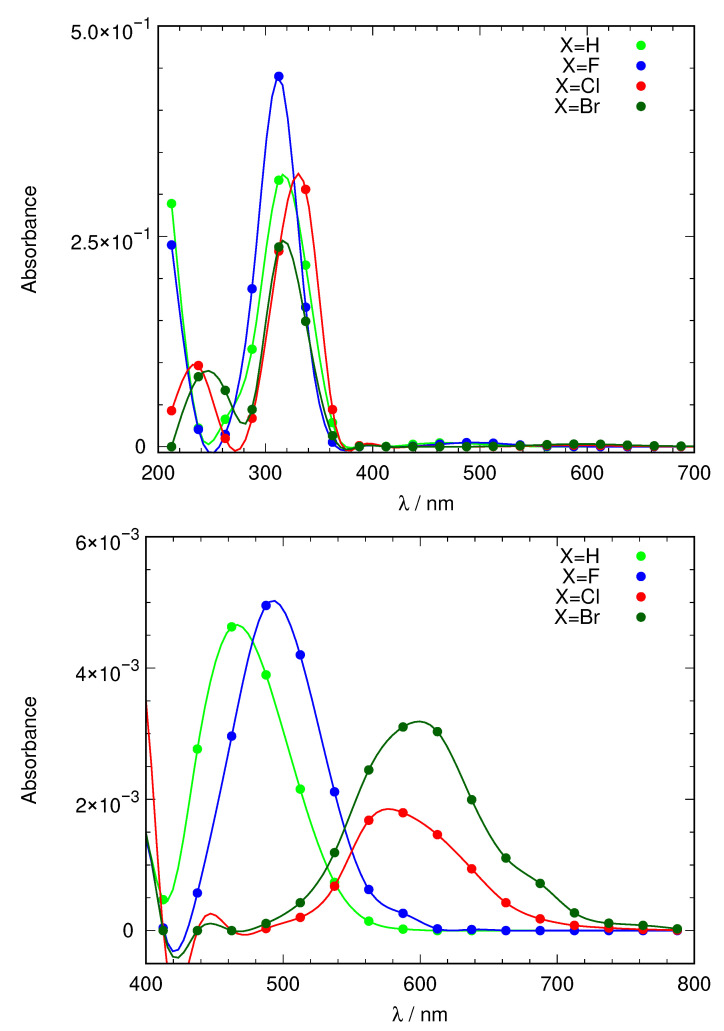
Simulation of the absorption spectra of azobenzene derivatives (X=H, F, Cl, Br) based on a 30 ns QM/MM molecular dynamics simulation where the solute is described using DFTB. Top: full range including UV and visible spectra; Bottom: visible part of the spectrum highlighting the weak transition to $S_{1}$. Absorption is given in arbitrary units.

Figure 10 shows the simulated spectra for X=H, F, Cl and Br, derived from the 30 ns QM/MM MD simulation where the solute is described using DFTB. The weak band, $S_{1}$, approximately reproduces the trend shown in Table 3, but it includes the breadth originating in the plethora of geometries that the solute molecule explores in the simulation at constant temperature and pressure. The maxima of the bands are found approximately at the following values: H 460 nm, F 490 nm, Cl 570 nm, and Br 600 nm. However, the spread of the bands is noticeable, and in the case of the chlorine derivative, there is noticeable intensity some 80 nm on both sides of the maximum of the band. With all due precautions, the bromine compound is expected to be excitable using visible radiation of at least 30–50 nm longer wavelength than the chlorine counterpart, well inside the mammal optical window. Interestingly, the vertical excitation energy from the energy minimum in the ground electronic state in DMSO (described using CPCM) in the case X=Br is 462.5 nm, a transition with a negligible probability, as seen in Figure 10.

This study indicates that the bathochromic shift originates in an upward trend in the departing MO of the solute (which in this case is the HOMO) and not in the receiving MO. It is possible to correlate the energy of this orbital with the energy of the highest orbitals of the atom X.

As mentioned above, optimal use for each azobenzene photoswitch requires that the wavelength of the light be redshifted as much as possible within the corresponding absorption band. Therefore, a green light irradiation at 527 nm is typically used experimentally for the *E-Z* isomerization in tetra-*ortho*-fluoroazobenzenes [3]. Note that this value is near the limit of the red side of the absorption band of the tetra-*ortho*-fluoroazobenzene predicted by our calculations (Figure 10). In turn, red light between 630 and 660 nm is used for the *E-Z* isomerization in tetra-ortho-chloroazobenzenes [3,13]. Compared with our calculated bands, shown in Figure 10, this irradiation range appears clearly outside the band corresponding to tetra-*ortho*-fluoroazobenzene, but it is now close to the edge of the red side of the absorption band of the tetra-*ortho*-chloroazobenzene. We think that this agreement validates the calculated absorption bands obtained here and the method we have used to generate them.

## 3. Materials and Methods

### 3.1. Single Molecule Calculations

Single molecule determination of geometries and energies were performed with the Gaussian 16 software suite [27]. Electronic energies in the ground state were determined using density functional theory (DFT) using the long-range corrected functional CAM-B3LYP for closed-shell molecules and the unrestricted version for isolated atoms [28]. This hybrid functional avoids the problems of non-long-range-corrected functionals (such as B3LYP) that grossly underestimate the excitation energies of excited states of internal charge-transfer type [28]. Recent benchmarks analyzing a large set of molecules confirm CAM-B3LYP to be among the best functionals to accurately measure vertical excitation energies [29,30]. The 6-311+G(d,p) triple-ζ basis set was used throughout [31,32]. Calculations in solution for single molecules were carried out within the C-PCM polarizable continuum method [33,34] using DMSO as solvent, as implemented in the Gaussian 16 software suite. In all cases, energies of ground state structures were computed after optimization to a minimum and verification that all eigenvalues of the Hessian matrix were positive. Excitation energies of single molecules, either in gas phase or in a continuum reproducing the solvent, were computed using the time-dependent density functional theory (TD-DFT) with the same functional and basis set on the geometry of the corresponding minimum.

### 3.2. Molecular Dynamics Simulations

Two different kinds of molecular dynamics simulations were used in this work: pure molecular mechanics simulations using the generalized amber force field version 2 (GAFF2) [35] and quantum mechanics/molecular mechanics (QM/MM) molecular dynamics simulations using the density functional with tight binding (DFTB) method for the QM part. Parameterizations are described ahead in the corresponding subsections. In this work, molecular dynamics simulations were carried out using the amber program [36].

#### 3.2.1. Parameterizations

A few simulations were carried out using molecular mechanics (MM) by means of the GAFF2 force field. In this case, which includes the solvent (DMSO), the parameterization started by carrying out single-molecule optimization of the molecule using the method described in the section above on single molecule calculations. The program AnteChamber, implemented in Amber 2021 [36], was used to derive the partial charges and build the GAFF2 description of the molecule.

Other simulations were carried out using the quantum mechanics/molecular mechanics multilevel approach. In this case, the solvent (DMSO) was described using the GAFF2 force field parameters determined as explained above, while the solvent was included using the DFT Tight Binding approximation (DFTB) [37] with the general purpose DFTB3 parameter set appropriate for organic molecules [38,39]. The effect of the solvent molecules was introduced using the electronic embedding (EE) scheme.

In all cases, the simulations were carried out under periodic boundary conditions.

#### 3.2.2. Solvent Cell

A solvent box of DMSO molecules in the shape of a capped octahedron (2000 molecules in total) was equilibrated in two steps: first a short equilibration of 100 ps was carried out at constant volume and temperature, followed by a dynamics of 1 ns at constant temperature and pressure. Temperature was kept at 300 K, and pressure at 1 bar under periodic boundary conditions. Mean energy, kinetic temperature and density of the DMSO cell were monitored to verify that they were stable and free of drift. Density of the solvent at the end of the equilibration run was stable at 1.12 g/mL, which compares well to an experimental value of 1.1 g/mL. This cell was used for all runs involving solvent.

#### 3.2.3. MD Production Runs

Solute molecules were simulated either using MM (GAFF2 force field) or QM/MM methodology (using DFTB for the QM part). Either way, a single solute molecule was solvated using the equilibrated solvent box and subject to a short 1.5 ns equilibration at constant temperature (300 K) and pressure (1 bar) under periodic boundary conditions. Kinetic temperature, total energy and density were monitored and verified to remain constant at the expected values. After this re-equilibration, the system was considered ready for production.

Production runs where the solute was treated using pure MM methodology were limited to only 10 ns. Production runs where the solute was described using DFTB were run for a total of 30 ns in three batches of 10 ns. In all cases, the production runs were run keeping the temperature and pressure constant at the equilibrated values.

### 3.3. Spectrum Simulations

A number of snapshots were sampled from the MD simulations computed, amounting to one snapshot every 10 ps to guarantee that these were not correlated. As the solute molecules can (and do) wander around in the unit cell used to run the dynamics, the snapshot was reconstructed with the solute at the center of the cell. The resulting structure was used to carry out a single molecule (the solute) electronic structure calculation subject to the electric field generated by the solvent molecules in the specific configuration of each snapshot, as derived from the GAFF2 charges of the atoms of each DMSO molecule in the actual positions of its nuclei. A fixed geometry calculation of the excitation energies of the 10 lowest singlet excited electronic states of the solute molecule using the *massage* option in Gaussian 16 was used to introduce the solute polarization caused by the solvent molecules, in this case represented by the partial charges of their atoms in their corresponding positions.

The oscillator strength (*f*) associated to the electronic transition between two electronic states *i* and *f* is given by:

(4) $$f_{if}=\frac{2m_{e}}{3\hbar^{2}e^{2}}\Delta E_{if}{\left|\mu_{if}\right|}^{2}$$

where $\mu_{if}$ is the transition dipole moment associated to the transition between states *i* and *f*. Its square modulus is proportional to the probability of the transition, so it can be written that the transition probability is given by

(5) $$P_{if}\propto \frac{f_{if}}{\Delta E_{if}}.$$

It is possible to build up a simulation of the absorption spectrum of the sample at room temperature by collecting the list of excitation energies and oscillator strengths of all excitations and all snapshots (obtained at the given temperature) and building a histogram of excitation energies $\Delta E_{0f}$ (0 denotes the ground electronic state) where each contribution adds $f_{if}/\Delta E_{if}$ (instead of one as in a regular frequency histogram). The histogram derived in this way is proportional to the probability of transition at each excitation energy and can be taken as a simulation of the absorption spectrum.

## 4. Conclusions

In this paper, an application of the “ensemble method” was used to compute the full shape of the absorption spectrum of a family of compounds based on the azobenzene basic building block. These molecules are useful photoswitch candidates with potential uses in fields, such as photopharmacology and optogenetics, where a critical design goal is the ability of the photoswitch to interact with radiation in the so-called mammal optical window (roughly 650–1100 nm, where mammal tissue is most transparent and affords deep penetration of light). The usual procedure to predict the position of the maxima of the absorption (and even emission) spectra is based on the Franck–Condon principle applied to the geometry of the minimum in the initial electronic state. However, these photoswitches have been experimentally known to be activated at wavelengths distant from their peak of absorption. This happens when either the minima of both ground and excited electronic states are quite different or when thermal contributions populate an extensive configurational region in the ground electronic state that is relevant to spectroscopy. This is the case of the azobenzene derivatives studied in the present work. As a matter of fact, we found that the vertical excitation energy from the energy minimum in the ground electronic state can have a weak transition probability with negligible contribution to the electronic absorption band.

We used MD simulations to explore the relevant regions of configurational space for these photoswitches in solution. A large number of snapshots of the system were sampled and used to compute excitation energies of the photoswitch along the MD simulation. These data were used to build a reasonable reproduction of the absorption spectrum of these photoswitches. In this way, it was possible to reproduce the two main features in the low-energy UV-Vis spectrum of the *E* isomers of azobenzene and its tetra-*ortho*-fluorinated and -chlorinated derivatives: a strong band in the UV region of the spectrum that was identified with a $\pi \pi^{*}$ transition (around 320–340 nm) and a weak absorption band peaking around 500–600 nm, described as a $n\pi^{*}$ transition. In both cases, the position and rough spread of the band was accounted for.

Focusing on the least energetic transition (to $S_{1}$, the one most amenable for the uses in living tissue), the energy of the orbitals involved was analyzed, and it was found that the energy of the orbital where the excitation starts in the azobenzene molecule correlates strongly with the energy of the highest occupied AO of the halide atom in question. The increase of energy of this molecular orbital was detected as the main cause for the bathochromic shift of the $n\pi^{*}$ band in these compounds. This observation suggested a different compound as a test case where to check our findings: the brominated derivative. Bromine has its highest occupied atomic orbital about 1 eV higher in energy than chlorine. We confirmed the viability of this mechanism computationally by repeating the procedure to construct the spectrum, this time with bromine as substituent. It has to be emphasized that the $n\pi^{*}$ band for the cases of the chlorinated and brominated derivatives penetrated into the mammal optical window.

MD simulations using adequate force-fields or better, suitable and affordable QM methods such as DFTB (used in this work), combined with excitation energy calculations on a large set of snapshots obtained from the MD simulation have proven an affordable way to obtain the general line shape of the spectrum.

The identification of the energy level of the free halide atom as a factor having a strong influence on the energy gap in the resulting photoswitch provides a useful insight into the operation of these molecules, and in the end, a practical criterium to design bathochromically shifted variants of these molecules.

## Figures and Tables

**Table 1:** Vertical excitation energies, excitation wavelengths and oscillator strengths of the two lowest singlet electronic excited states of the *E* tetra-*ortho*-substituted azobenzenes in gas phase and in DMSO.

	$S_{1}$			$S_{2}$		
Gas	Δ *E*	λ	f	Δ *E*	λ	f
	eV	nm		eV	nm	
X=H	2.753	450.3	0.000	4.112	301.6	0.779
X=F	2.680	462.6	0.037	4.525	274.0	0.710
X=Cl	2.685	461.8	0.025	4.512	274.8	0.025
	$S_{1}$			$S_{2}$		
**DMSO**	ΔE	λ	f	ΔE	λ	f
	**eV**	**nm**		**eV**	**nm**	
X=H	2.768	448.0	0.000	3.936	315.0	0.915
X=F	2.698	459.6	0.053	4.249	291.8	0.891
X=Cl	2.716	456.5	0.032	4.438	279.4	0.043

**Table 2:** Approximate weights of the AOs located on the N and X (X=F, Cl and Br) atoms of the solute in the MOs involved in the excitation to $S_{1}$, which in all cases are the HOMO (as origin of the excitation) and LUMO (as its destination). The ω values given correspond to the vertical excitations computed at the gas phase minimum energy structure in the ground electronic state using Equation (3).

	HOMO		LUMO	
	$\omega_{N}$	$\omega_{X}$	$\omega_{N}$	$\omega_{X}$
X=F	0.41	0.10	0.51	0.10
X=Cl	0.43	0.19	0.49	0.13
X=Br	0.42	0.22	0.56	0.12

**Table 3:** Average excitation energies, average energies of the HOMO and of the LUMO involved in the excited $S_{1}$ state in the 30 ns DFTB/MM MD simulation.

X	〈ΔE〉/eV	$\langle E\left({\mathrm{MO}}_{i}\right)\rangle$/eV	$\langle E\left({\mathrm{MO}}_{f}\right)\rangle$/eV
H	2.645	–8.357	–1.606
F	2.509	–8.376	–1.774
Cl	2.114	–7.920	–2.003
Br	2.091	–7.673	–1.939

## Data Availability

The data presented in this study are contained within the article.
